# A guide for researchers seeking training in retrospective data harmonization for population neuroscience studies of Alzheimer’s disease and related dementias

**DOI:** 10.3389/fnimg.2022.978350

**Published:** 2022-09-26

**Authors:** C. Elizabeth Shaaban, Dana L. Tudorascu, M. Maria Glymour, Ann D. Cohen, Rebecca C. Thurston, Heather M. Snyder, Timothy J. Hohman, Shubhabrata Mukherjee, Lan Yu, Beth E. Snitz

**Affiliations:** 1 Department of Epidemiology, School of Public Health, University of Pittsburgh, Pittsburgh, PA, United States; 2 Department of Psychiatry, School of Medicine, University of Pittsburgh, Pittsburgh, PA, United States; 3 Department of Epidemiology and Biostatistics, School of Medicine, University of California, San Francisco, San Francisco, CA, United States; 4 Medical and Scientific Relations, Alzheimer’s Association, Chicago, IL, United States; 5 Vanderbilt Memory and Alzheimer’s Center, Vanderbilt University Medical Center, Nashville, TN, United States; 6 Department of Medicine, University of Washington, Seattle, WA, United States; 7 Department of Medicine, School of Medicine, University of Pittsburgh, Pittsburgh, PA, United States; 8 Department of Neurology, School of Medicine, University of Pittsburgh, Pittsburgh, PA, United States

**Keywords:** neurocognitive testing, neuropsychological assessment, cognitive aging, pooling data, neuroimaging, sample size, external validity

## Abstract

Due to needs surrounding rigor and reproducibility, subgroup specific disease knowledge, and questions of external validity, data harmonization is an essential tool in population neuroscience of Alzheimer’s disease and related dementias (ADRD). Systematic harmonization of data elements is necessary to pool information from heterogeneous samples, and such pooling allows more expansive evaluations of health disparities, more precise effect estimates, and more opportunities to discover effective prevention or treatment strategies. The key goal of this Tutorial in Population Neuroimaging Curriculum, Instruction, and Pedagogy article is to guide researchers in creating a customized population neuroscience of ADRD harmonization training plan to fit their needs or those of their mentees. We provide brief guidance for retrospective data harmonization of multiple data types in this area, including: (1) clinical and demographic, (2) neuropsychological, and (3) neuroimaging data. Core competencies and skills are reviewed, and resources are provided to fill gaps in training as well as data needs. We close with an example study in which harmonization is a critical tool. While several aspects of this tutorial focus specifically on ADRD, the concepts and resources are likely to benefit population neuroscientists working in a range of research areas.

## Introduction: Background and rationale

Neuroscience studies, including those focused on Alzheimer’s disease and related dementias (ADRD), are often marked by small sample size and highly selective participation. Consequently, many studies do not represent diversity with respect to race/ethnicity, age, comorbid conditions, education, income, or geographic factors, limiting the population relevance of the research. Further, study participation may be influenced by complex combinations of these or other variables such as gender/sex or genetic characteristics.

These selection processes create critical limitations to the quality of the neuroscientific evidence base. Work in the Adolescent Brain Cognitive Development Study (*N* = 11,878) has demonstrated that small sample sizes reduce reproducibility of study findings. Investigators showed that brain-behavior correlations in brain-wide association studies can differ, not only in strength, but worryingly in directionality. Across multiple draws of small sample size the direction of associations may be reversed, but results are more reproducible at large sample sizes (Marek et al., 2022). This has negative implications for both reproducibility and pooled and meta-analyses. Small, highly selected samples also reduce statistical power to test for subgroup effects (e.g., women vs. men; *APOE4* carriers vs. non-carriers; those with vs. without cardiovascular risk factors). Evaluating effects within subgroups, and estimating differences in effects across groups, is essential to determine how to tailor interventions to prevent neurocognitive decline and identify drivers of brain health inequalities.

Increasing sample size is important but cannot fully rectify these limitations; attention to other features of rigorous and robust research designs is needed. First, to obtain larger sample sizes, researchers may wish to combine multiple data sets. In population neuroscience studies, attempts to pool neuroimaging data collected across scanners, sites, and cohorts will be subject to “scanner effects” (Fortin et al., 2018)—technical sources of variance. Scanner effects have been reported in multiple neuroimaging measures derived from MRI and PET (Fortin et al., 2016, 2017, 2018; Yu et al., 2018; Pomponio et al., 2020). Even in multi-center studies using good principles of study design for prospective harmonization, scanner or site-related factors can explain large proportions of variance in the neuroimaging measure (Shinohara et al., 2017). Scanner or site effects, unless corrected, introduce error variance, reducing power to detect effects of interest such as sex interactions (Leek and Storey, 2007). Furthermore, scanner differences likely become associated with study population and compositional differences, rendering this research vulnerable to potential confounding bias. Thus, not accounting analytically for scanner effects can be consequential.

Second, when there is effect modification of an association between an exposure and an outcome, external validity will be impacted by differing prevalence of effect modifiers in the sample vs. the target population (Cole and Stuart, 2010; Keyes and Westreich, 2019). An illustration of this phenomenon can be seen in a comparison of associations in the highly selected Alzheimer’s Disease Neuroimaging Initiative (ADNI) study and the community-based Atherosclerosis Risk in Communities (ARIC) study (Gianattasio et al., 2021). Compared to ARIC participants, ADNI participants were more likely to male, *APOE4*+, married, more highly educated, to have mild cognitive impairment (MCI) or dementia, and less likely to be Black or have a history of hypertension (Gianattasio et al., 2021). Approximately 1/3 of associations varied significantly by study, and some effect size differences were *very* large (e.g., odds ratio for association of *APOE4* with (Aβ)+ = 8.6 in ADNI but 2.8 in ARIC), likely due to differences in prevalence of effect modifiers between the two studies (Gianattasio et al., 2021).

**FIGURE 1:**
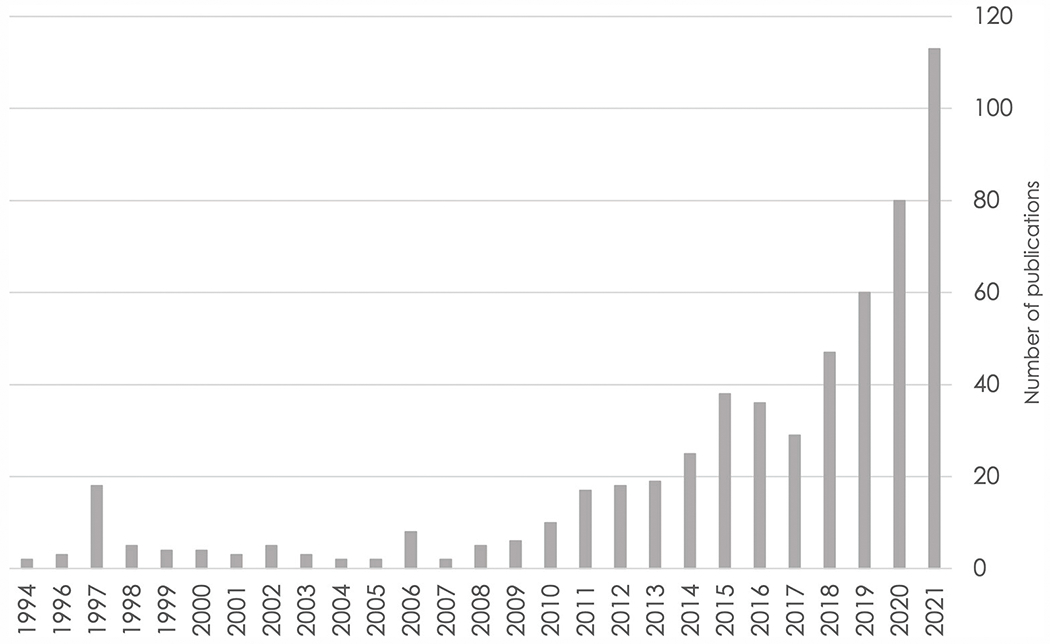
Number of publications over time based on a PubMed search for cognitive aging and Alzheimer’s disease and related dementias through the end of 2021. Search query: ((((alzheimer’s disease) OR (dementia)) OR (mild cognitive impairment)) OR (cognitive aging)) AND (harmonization).

Data pooling underlies the ability to address each of the limitations described above, but data cannot be pooled without careful harmonization. As data sharing of numerous neuroscience datasets is becoming more common, the number of publications using harmonization to study brain health is increasing (see Figure 1 for an example from the cognitive aging and ADRD literature). Thus, harmonization is now an essential skill for population neuroscientists.

Harmonization “refers to all efforts to combine data from different sources and provide users with a comparable view of data from different studies” (Data Sharing for Demographic Research, 2022). The aim is to synthesize data to render it similar enough to either be (1) combined for pooled data analysis or (2) analyzed in parallel in the same manner and compared (e.g., reproducing an analysis carried out in one study sample within a different study sample). A “stringent” approach to harmonization involves multiple studies agreeing in advance of data collection to use the same assessments and protocols to prospectively collect the same data (Fortier et al., 2011). On the other hand, a more “flexible” harmonization approach allows for differing assessments and study protocols (Fortier et al., 2011). This approach may be carried out prospectively, but also allows for retrospective harmonization. We focus our comments in this paper specifically on retrospective data harmonization so that early career researcher (ECR) population neuroscientists and others new to the field develop training to make efficient and accurate use of existing data. The audience and objectives of this article are detailed next.

## Audience, environment, objectives, and outcomes

Approaches to retrospective harmonization vary, and there exist few sources of integrated guidance addressing the varied data types that population neuroscientists commonly use. We aim to address how new researchers can obtain training in this area with this introductory level Tutorial in Population Neuroimaging Curriculum, Instruction, and Pedagogy article. The article is directed to ECRs (students, postdocs, and early career faculty) and others new to the field as well as faculty teaching related courses and mentoring trainees. This information would be especially relevant for researchers writing government and foundation funded training grants. While the example (section Example research plan incorporating population neuroscience of ADRD harmonization) and many data sources in Table 2 are ADRD-specific, population neuroscientists across the life course and health and disease states are likely to benefit from this tutorial. The learning approach and environment are highly self-directed and based upon a mentored academic model in which the trainee works with mentors to identify gaps in knowledge and training elements to fill those needs and build their skills.

The objectives of this article are to (1) illustrate for researchers, reviewers, and funders the need for population neuroscience data harmonization (Introduction); (2) describe core competencies and skills necessary for harmonization methods of the data types that population neuroscientists should have expertise in; (3) assist readers in identifying their own training gaps and list a selection of relevant learning resources; and (4) use an example ADRD research question to further examine considerations in harmonization of demographic/clinical, neuropsychological, and neuroimaging data. Overall, the expected learning outcome of this article is the creation of a customized population neuroscience of ADRD (Ganguli et al., 2018) harmonization learning plan to fit readers’ needs or those of their mentees. Evaluative feedback on the developed training plan can be carried out through an iterative process of mentor feedback and revision, while the final evaluation for those writing training grants will be in the form of peer review and feedback on the grant to the investigator. Key elements for positive evaluation are the extent to which the training resources selected by the researcher map onto knowledge gaps and the extent to which the training is integrated into and necessary to answer the researcher’s scientific questions.

## Brief introduction to harmonization and core competencies and skills

Detailed guidance regarding overall retrospective data harmonization is provided in the Maelstrom Research Guidelines (Fortier et al., 2017). In this section, we provide a brief introduction to the Maelstrom best practices and related core competencies and skills researchers need to carry out rigorous harmonization in population neuroscience studies. Core competencies and training resources to address researcher knowledge gaps are outlined in Table 1. We recommend that researchers use this table and work with their mentors to identify which core competencies are training gaps (Table 1, left column) and design their own training plan based on the resources provided (Table 1, right column). This should be an iterative process of design and drafting, mentor feedback, and training plan revision. This is a key component in career development award proposals. Data resources for ADRD studies are provided in Table 2. These may be sources of data for use in researcher harmonization studies, additional training information, and in some cases, small grants may be available. Some of the data sources listed are highly selected samples/cohorts [e.g., ADNI and the National Alzheimer’s Coordinating Center (NACC)], and population neuroscientists are encouraged to evaluate external validity more formally when these samples are used.

Population neuroscience entails a convergence of expertise in epidemiology and neuroscience/neuroimaging (Paus, 2010; Falk et al., 2013). If harmonization is to be undertaken in large epidemiological cohorts with neuroimaging, the population neuroscientist must be skilled in harmonizing demographic and clinical data as well as neuropsychological and neuroimaging data. Next, we provide an introduction to harmonization, core competencies, and necessary skills both overall and for the specific data types population neuroscientists will encounter.

### Overall harmonization

Retrospective population neuroscience harmonization requires expertise across a range of disciplines. As such, multidisciplinary collaboration skills are critical. Pre-statistical harmonization ensures rigorous, high quality research results, and includes selection of appropriate studies and variables to incorporate in the harmonization. This involves creating the DataSchema—the list of variables needed to answer the specific study question—and assessing these variables in each study for harmonization potential (Fortier et al., 2017). The DataSchema includes the key predictors(s), outcome(s), confounders, and effect modifiers of interest. The research question, population studied, and necessary data on exposures, outcomes, and other key variables should dictate which studies are selected for harmonization. Detailed documentation of each contributing study’s design characteristics and variables will be needed for study selection and assessment of variable compatibility with the DataSchema and harmonization potential. This process will require expert input. Specifically, the following study design information should be documented:

- Is the study population representative or volunteer based? Who is the target population for the sample if the population is representative, and if not, who is in the sample?
- What was involved in study participation? Were there different modalities or degrees of participation (e.g., home visit vs. clinic visit)? What were predictors of participation, if known?
- How were measurements conducted? Are there alternative sources of information about people who did not complete the measurement?

Next, data will be requested from contributing studies and transformations of available variables into a common data format will be applied with statistical analysis software. All decisions regarding transformations will need to be documented. If it is unclear how multiple variables can be mapped onto a final harmonized version, referring to NIH’s common data element (CDE) repository (https://cde.nlm.nih.gov/) or the Gateway to Global Aging Data site’s (https://g2aging.org/documentation) data documentation may give helpful starting points. Throughout the harmonization and analysis process, we recommend the use of GitHub (https://github.com/) paired with code review by another team member (Vable et al., 2021) to promote transparent, reproducible statistical analysis. More details about how to incorporate these features into a cognitive data harmonization workflow can be found in Mukherjee et al. (2022), but are applicable to all data types reviewed in this Tutorial.

Statistical coding and analysis skills will be needed when: running power or sample size calculations to confirm the study is appropriately powered; assessing variable distributions, missingness, harmonized data quality, and representativeness; transforming variables; using imputation or latent variable-based harmonization approaches for neuropsychological data; weighting to address selective participation; and carrying out primary and sensitivity analyses to test the major harmonized variables being used in the analyses.

Accurate, transparent reporting is needed when (1) reporting back to original contributing studies, (2) publishing harmonized study results, and (3) providing harmonized data to future users.

Finally, researchers will need to learn effective project leadership and respectful partnership with stakeholders while requesting and working on data and detailed documentation relating to the project (Fortier et al., 2017; Lesko et al., 2018). Additional guidance regarding decisions relevant to these skills in harmonization and pooled analyses may be found in Lesko et al. (2018).

### Clinical and demographic variable harmonization

Clinical and demographic variable harmonization requires domain expertise regarding how to define and collapse across categories. Because flexible, retrospective harmonization requires that data be combined in a way that supports “inferential equivalence” (Fortier et al., 2011), researchers will need to assess which variables cannot be combined due to compromised measurement validity. For example, consider alternative approaches to assessing prevalent hypertension, a common and nominally straightforward risk factor:

- Self-reported response to “Has your doctor ever told you that you have high blood pressure or hypertension?”
- Selecting hypertension from a list when instructed “Have you ever been diagnosed with any of the following conditions? Please select all that apply.”
- Hypertension recorded in medical records before or after practice guidelines changed in 2017.
- Hypertension based on study measurements of blood pressure.

Can hypertension measured in these heterogeneous ways across studies be conceptualized as the same variable? What is the sensitivity and specificity of each measure for prevalent hypertension and how will the misclassification in each study impact findings? Are there possible pre-processing steps to make the measures more comparable, or bias corrections to reduce the impact of misclassification analytically? This will also require content knowledge about the construct being measured, review of the literature on measurement characteristics of each approach, and good documentation from the studies to know how the measurement was conducted.

### Neuropsychological assessment harmonization

Neuropsychological assessment harmonization requires expertise regarding cognitive domains and processes, knowledge of testing protocols and standards, as well as relevant analytic competencies such as descriptive statistics, data visualization, and variable transformations. Different harmonization strategies include linear transformations/standardization (such as z-scores), equipercentile equating, multiple imputation approaches, and psychometric and latent variable techniques including factor analysis and item response theory (IRT).

Standardization methods, when used to enable data pooling, impose strict assumptions and are only appropriate if the contributing data sets were all representative of the same population, or if populations are known to have the same distribution of the neuropsychological scores. This is rarely plausible because of the strong influence of cognitive function on study participation. Standardization methods can also create circularity when comparing studies. For example, if the impairment definition is based on the within-study distribution of cognition, the prevalence of impairment will be identical for all studies.

Z-scores are a commonly used approach in ADRD research. However, there are several cautions regarding their use. While averaging all z-scored tests within a cognitive domain is an often-used approach to obtain a domain score, this simply puts test scores on the same scale, but has not harmonized them absent confirmation that the tests equivalently measure the underlying domain of interest. Aside from making the distributional assumption mentioned above, simple average domain z-scores assume equal test contribution within domains (e.g., a memory domain z-score comprised of four test z-scores assumes that each test makes up 25% of the memory domain). Some of these problems of sum and mean scores have been recently reviewed (McNeish and Wolf, 2020), and a recent paper illustrates some of these principles by moving from a preclinical Alzheimer cognitive composite (PACC) z-score to a harmonized PACC using IRT (Hampton et al., 2022). A final caution on z-scores is that many neuropsychological assessment batteries change over time, and z-scoring cannot provide inferential equivalence under this circumstance.

Equipercentile equating preserves rank across two assessments, determines the score on one assessment that is equated to the score on the other, and the equated score can then be imputed as the value for the assessment of interest. An example application in ADRD research addressed changing neuropsychological batteries in the NACC Uniform Data Set (UDS), equating the Mini-Mental State Examination (MMSE, Folstein et al., 1975) with the Montreal Cognitive Assessment (MoCA, Nasreddine et al., 2005; Monsell et al., 2016). This approach is only applied when an a priori level of correlation between the two assessments is achieved (here, a correlation coefficient of ≥0.6). The sample can be divided into a training set to develop the equating and a test set to test the accuracy.

Multiple imputation and IRT do not rely on the population distributional assumption of standardization, but instead rely on the availability of at least one, but ideally many, items that are identical across samples. Multiple imputation relies on the assumption that the associations between items are identical across studies. In multiple imputation approaches, an assessment which was not completed in one study but was completed in others is treated as systematically missing in the stacked dataset. The missing data is then imputed. One recent approach in ADRD has used a random forest model to learn the association between the neuropsychological assessment of interest (non-missing) with all other variables in the dataset and then imputes the missing values of the assessment of interest based on that structure (Shishegar et al., 2021). A starting value for the missing data is preselected and entered into the model. The model then outputs an estimate for the missing data and those initial and new values are compared. This iterative process stops when a predetermined difference between the initial and new values meets a predetermined, sufficiently low threshold.

Item response theory offers methods to assess the assumption that tests (referred to as test *items* in latent variable modeling) are equivalent across studies and estimate latent variables even under modest violations of that assumption. Item response theory models are thus more flexible and rigorous because they build in methods to assess harmonization validity. However, IRT methods still rely on the availability of at least some truly equivalent items. We describe latent variable approaches in detail in Section Neuropsychological assessment harmonization. This approach has been recently detailed in ADRD research in the following publications (Mukherjee et al., 2020, 2022). Suggested readings on these topics and cross-national harmonization approaches are listed in Table 1.

### Neuroimaging harmonization

Population neuroscientists in ADRD research should know that several neuroimaging harmonization approaches exist. For example, standardization approaches of interest include binarization and the Centiloid scale. Many studies make use of binarization to determine positivity or negativity on some biomarker of interest, e.g., Aβ, tau, and neurodegeneration. The limitations of this approach are that it does not deal with processing pipeline differences or scanner effects, loses information from the continuous version of the variable, and allows for only coarse longitudinal change tracking (Lesko et al., 2018). The Centiloid scale is a standardization approach to put different amyloid PET tracers on the same scale, allowing data pooling across tracers (Klunk et al., 2015). The Centiloid scale is framed by 0–100; the 0 anchor represents high-certainty amyloid negative cases, i.e., amyloid level in the brains of healthy young controls (≤45 years of age), and the 100 anchor reflects amyloid level in the brains of typical AD dementia patients. Because these anchor points are averages, the full range of the scale can run from below 0 to >100. Transformation equations and amyloid positivity cut points across tracers for crosssectional and longitudinal analyses have been suggested (Royse et al., 2021). Other approaches such as non-linear distributional mapping (NoDiM) do not assume linearities in amyloid PET tracer measurement scales (Properzi et al., 2019).

There are regression-based statistical harmonization approaches which can be applied either pre- or post-image processing. These methods include RAVEL (Removal of Artificial Voxel Effect by Linear regression, Fortin et al., 2016) and ComBat [combatting batch effects when combining batches of gene expression microarray data (Fortin et al., 2018), with its original use in gene expression data]. Also important is the ability to combine multiple approaches when building neuroimaging and data processing pipelines, such as our own pipelines from MRI pre-processing harmonization using RAVEL to inform PET quantification (Minhas et al., 2020) and approaches incorporating RAVEL and ComBat in the same pipeline to address both MRI image intensity and other scanner effects (Torbati et al., 2021a). We review RAVEL and ComBat in more detail in Section Neuroimaging harmonization.

Finally, machine learning-based neuroimaging harmonization approaches include MISPEL (Multi-scanner Image harmonization via Structure Preserving Embedding Learning), an approach to MRI harmonization developed for use with more than two scanners (Torbati et al., 2021b). DeepHarmony and mica are harmonization approaches that address MRI contrast when two scanners (Dewey et al., 2019) or more are used (Wrobel et al., 2020).

Researchers are advised to note that varying imaging processing softwares and versions are another source of unwanted noise when pooling neuroimaging data (Tudorascu et al., 2016). The strongest approach for dealing with this problem would be to process all images with the same program and version pipeline, and program and version should always be reported. Suggested readings on harmonization approaches dealing with cross-sectional and longitudinal MRI, PET, and new and combined pipelines are provided in Table 1. To illustrate an example of retrospective data harmonization in a population neuroscience of ADRD study, next we describe the workflow of a planned study on sex differences in the AD biomarker cascade.

## Example research plan incorporating population neuroscience of ADRD harmonization

### Background and study aim

Men and women may differ in pathways to AD with critical implications for personalized interventions. Women are consistently found to have more tau accumulation in the brain than men after accounting for age (Filon et al., 2016; Hohman et al., 2018; Oveisgharan et al., 2018; Buckley et al., 2019a,b, 2020; Luchsinger et al., 2020; Edwards et al., 2021; Palta et al., 2021), with few studies reporting no differences or reverse directionality (Morris et al., 2010; Altmann et al., 2014; Buckley et al., 2019a; Ziontz et al., 2019). Some studies find women also have more brain Aβ than men (Barnes et al., 2005; Jack et al., 2015; Hohman et al., 2018; Liesinger et al., 2018; Oveisgharan et al., 2018; Sundermann et al., 2018; Buckley et al., 2019b; Luchsinger et al., 2020; Rahman et al., 2020; Edwards et al., 2021; Palta et al., 2021), though others do not (Morris et al., 2010; Mielke et al., 2012; Altmann et al., 2014; Filon et al., 2016; Buckley et al., 2018; Sperling et al., 2020; Edwards et al., 2021; Yan et al., 2021). In addition, the relationship between Aβ and tau may vary by sex (effect modification), with this relationship being stronger in women than men (Buckley et al., 2019b, 2020).

In addition to Aβ and tau, cerebral small vessel disease (cSVD), pathology of the small arteries, veins, and capillaries of the brain (Pantoni, 2010; Wardlaw et al., 2013, 2015), may be an important part of the pathway to AD (Kester et al., 2014; McAleese et al., 2015; Tosto et al., 2015; Lee et al., 2016, 2018; Debette et al., 2019; Greenberg et al., 2020), and late-life women have a greater burden and risk of cSVD than age-matched men (Longstreth et al., 1998; Uehara et al., 1999; Vermeer et al., 2002; van Dijk et al., 2008; Nyquist et al., 2014). Among those with clinical AD dementia and mixed pathology on postmortem exam, women are likelier than men to have mixed AD and cerebrovascular pathology (Barnes et al., 2019). Several pieces of evidence implicate vascular damage as an important part of AD pathophysiology. First, cerebrovascular disease and AD share risk factors (Dichgans and Zietemann, 2012; Jorgensen et al., 2018; Shaaban et al., 2019). Second, cerebrovascular dysfunction has been shown early in the transition from cognitively unimpaired (CU) to impaired, preceding Aβ deposition (Iturria-Medina et al., 2016). Finally, postmortem clinical-pathologic samples demonstrates that pure AD pathology in AD dementia cases is rare (~4%), while 87% have co-occurring vascular pathology (Kapasi et al., 2017). Therefore, a research and public health focus on modifiable vascular contributors to AD dementia is imperative (Gorelick et al., 2011; Snyder et al., 2015; Corriveau et al., 2016). Understanding the role of cSVD in sex differences in the AD pathophysiological cascade could reveal intervention targets and markers of target engagement which could be used to reduce AD dementia.

One specific aim of this study is to quantify sex differences in the cSVD-AD pathway. We will also explore associations of sex-related factors (e.g., pregnancy history, menopause, hormone use; signs and symptoms of hypogonadism) with cSVD, Aβ, and tau. In this study, we will harmonize data from five longitudinal cohort studies: PiB Normal Aging (Aizenstein et al., 2008), Heart SCORE A and B (neuroimaging sub-studies of the parent study, Heart Strategies Concentrating on Risk Evaluation; Snitz et al., 2020), MYHAT-NI (a neuroimaging sub-study of the parent study, Monongahela-Youghiogheny Healthy Aging Team; Sullivan et al., 2020), and Human Connectome Project (HCP)-Pitt (Cohen et al., 2021).

### Why is harmonization needed?

First, to detect sex differences and explore sex-specific relationships, we need a larger sample size than available in any individual study. Harmonization will allow us to standardize and pool data across the contributing studies and conduct joint analyses in the larger sample. Second, we would like to enhance the external validity of our estimates. Although all contributing studies have been carried out at the University of Pittsburgh and draw from the local southwestern Pennsylvania population, they were volunteer-based and not population-representative. The selection factors that led to women being included in a study sample likely differed from those operating in men; failing to account for these potentially gives a misleading picture of sex differences. Furthermore, cardiovascular risk factors and common comorbidities of aging (1) are important to consider in the cSVD-AD pathway; (2) are far more common in the population than in highly selected studies; and (3) may vary in prevalence by sex. Since our long-term goal is to improve brain health on the population level, addressing these threats to external validity is crucial to understanding whether sex differences exist at the level of various populations of interest.

Harmonization will allow us to standardize. Weighting and other methods will allow us to adjust the estimates from our study sample to those we should find in the local population. This is further described below in Section Harmonization for external validity analyses.

### A scientific caution

We caution the reader to be thoughtful about the capacity, within harmonization approaches, to remove differences in measures due to certain variables. For example, neuroimaging harmonization approaches can regress out differences due to sex. However, our primary scientific interest is in sex differences and sex-specific pathways, and therefore we wish to preserve the variance in our outcomes that is attributable to sex. The neuroimaging harmonization approaches we describe below can accommodate this if specified in the model. Such decisions must be made while designing the harmonization plan and are fully dependent on each specific scientific question.

### How will harmonization be carried out?

Harmonization will follow the procedures laid out in Sections Brief introduction to harmonization and core competencies and skills and Overall harmonization above, following Maelstrom guidance (Fortier et al., 2017). We will develop the DataSchema and assess for harmonization potential of the variables we have in the contributing studies by data type.

### Clinical and demographic variable harmonization

We will pool data including demographics, cardiovascular risk factors/common comorbidities of aging (e.g., hypertension, diabetes, congestive heart failure, obesity, smoking, and physical activity) and cognitive status based on content area expertise on the study team. Coding of these characteristics across cohorts will be documented and transformed as needed to develop a harmonized dataset. All cohort studies except MYHAT-NI adjudicate cognitive status yearly with a consensus conference modeled on the University of Pittsburgh Alzheimer’s Disease Research Center. Neurologists, psychiatrists, neuropsychologists, and other clinicians review medical history, medications, neurologic and psychiatric exams, neuropsychological testing, and neuroimaging. In MYHAT-NI, cognitive status is initially based on the Clinical Dementia Rating (CDR) (Morris, 1993) scale: cognitively unimpaired (CU), CDR = 0; MCI, CDR = 0.5; dementia, CDR ≥ 1. Etiologic diagnosis of all incident dementia cases in MYHAT-NI is then determined by a “virtual consensus conference” (Lee et al., 2020), with inter-disciplinary experts reviewing clinical data online and making etiologic diagnostic ratings.

### Neuropsychological assessment harmonization

All contributing studies administer detailed neuropsychological assessments yearly. We wish to conduct a rigorous harmonization to allow data pooling across contributing studies and the possibility of comparisons with other studies in the future. In our case, all contributing studies are recruited from the same overall geographic population, with many aspects of shared language and culture, and multiple equivalent neuropsychological test items across studies. Furthermore, our contributing studies are longitudinal, and some are longstanding with potential for changing test batteries over time. Item response theory best meets our needs for inferential equivalence across studies and over changing test batteries, and the data meet the requirements for IRT.

We will follow the rigorous IRT-based approach recently detailed in a cognitive harmonization workflow paper (Mukherjee et al., 2022) and initially developed for a genetics of late-onset AD across five studies (Mukherjee et al., 2020). We refer readers to Mukherjee et al. (2022) for detailed methods. This approach has been calibrated across the full spectrum of cognitive diagnoses from CU to AD dementia. Briefly, first, test administration and scoring procedures across contributing studies are fully documented to understand potentially important differences. Test variables are assigned to cognitive domains (memory, executive function, language, visuospatial abilities) by neuropsychologists. Next, we will assess data distributions, recoding reverse coded items, and missingness as part of the data quality control step. All transformations will be documented. We will confirm that the tests load onto their respective cognitive domain factors and obtain the best fitting model using confirmatory factor analysis. The scores will be co-calibrated to other studies with overlapping measurements, such as the Adult Changes in Thought study, NACC, the Framingham Study, and ADNI. Co-calibrations can be daisy-chained together, so even studies with no overlapping measurements with our study may still be compared, although each step introduces uncertainty in the measurement quality.

### Neuroimaging harmonization

The inter-visit interval for neuroimaging is 24 months in all contributing studies. MRI has been obtained across all studies using 3T Siemens scanners (1 TIM TRIO and 3 Prismas). ^11^C-PiB is used in all cohorts for Aβ PET, and 4/5 studies collect tau PET using ^18^F-AV-1451. All contributing study PET scans were conducted on either a Siemens Biograph mCT PET/CT or a Siemens/CTI ECAT HR+ PET. Our neuroimaging harmonization approach includes both pre- and post-processing regression-based harmonization methods, described below. The harmonized neuroimaging values are then used as the outcomes of interest in the overall substantive statistical analyses.

### Pre-processing neuroimaging harmonization

The images at all visits for the combined pool of participants (*N* = 870 cross-sectional and 645 longitudinal) will be preprocessed together as follows. We will use RAVEL (Fortin et al., 2016) applied to the T1 and FLAIR MRI scans. RAVEL incorporates two key steps that differ from typical MRI pre-processing pipelines. First, following typical segmentation into gray matter (GM), white matter (WM), and cerebrospinal fluid (CSF), transformation into a common anatomical space, and skull stripping, White Stripe image-intensity normalization (Shinohara et al., 2014) is applied. This is important because MRI intensity units are not standard across scanners, and this step removes variation due simply to arbitrary unit differences between visits and scanners. This approach z-scores voxel intensity based on the mean and standard deviation of intensity in normal appearing white matter (NAWM). Because of its large size, NAWM is less susceptible to partial volume effects and represents biologically healthy tissue. This processing is rapid—typically under 5 s per scan on a laptop (Shinohara et al., 2014). Second, a control region of interest (ROI) is identified (here, CSF) where image intensity should not vary as a function of AD or other biological variables of interest. Any variance seen in this ROI represents non-biological differences (e.g., scanner effects); this variance is regressed out in voxel-level linear regression. One advantage of RAVEL over other image intensity normalization methods is that it maintains variance due to biological factors of interest to study (e.g. sex, age, hypertension) by including them in the voxel-level regression (Fortin et al., 2016). This is critical as our primary interest is in examining sex differences. RAVEL is implemented in R statistical analysis software (R). After RAVEL is applied, the MRI is warped back to person-specific space, and in our pipeline, FreeSurfer software is applied to perform MR bias correction, automated ROI parcellation, and tissue segmentation. These harmonized MRIs will then be used for analysis of cSVD and to derive the PET ROIs according to our RAVEL to PET pipeline (Minhas et al., 2020).

### Post-processing neuroimaging harmonization

RAVEL harmonization may be more important for MRI markers than PET markers (Minhas et al., 2020). If an alternative PET harmonization approach is needed, we will apply post-processing statistical harmonization methods using ComBat (Fortin et al., 2018). In this case, regression-based harmonization is performed using PET SUVR based on FreeSurfer ROIs which have not been harmonized with RAVEL. Similar to the second step of RAVEL, it removes scanner effects while maintaining participant characteristic-related variance of interest when these variables are added to the harmonization model. ComBat is computationally efficient to use (Fortin et al., 2018) and can remove non-biological sources of variance when harmonized data acquisition protocols were not used (Fortin et al., 2018). It has been demonstrated to be effective when applied to multiple neuroimaging measures including GM volume, cortical thickness, diffusion tensor imaging, and fMRI (Fortin et al., 2017, 2018; Yu et al., 2018; Pomponio et al., 2020) and is implemented in R (Fortin, 2020).

### Harmonization for external validity analyses

We will leverage availability of data from a local countywide population-representative sample from the Behavioral Risk Factor Surveillance System (BRFSS; https://www.cdc.gov/brfss/). Carrying out these analyses requires that our overall ADRD dataset and the BRFSS dataset are stacked, thus requiring harmonization. We can then use variables that were measured in both the BRFSS and in our contributing ADRD studies to correct for selection (e.g., demographics and cardiovascular risk factors/common comorbidities of aging). For example, if men in the ADRD studies are much more likely to be married than men in the BRFSS, whereas for women marital status does not differ substantially, we can upweight unmarried men in our ADRD studies so the joint distribution of sex and marital status in our analyzed data matches the joint distribution in the population. We will use the two main G-methods for these external validity analyses: (1) inverse probability weighting (IPW) for sampling and (2) G-computation (Bareinboim and Pearl, 2016; Lesko et al., 2017; Westreich et al., 2017). This will allow us to adjust the estimates from our study sample and make them more generalizable to the target population.

## Conclusion

Studies of ADRD have proliferated and data sharing has increased and will be an NIH requirement as of January 2023 (see NIH Policy for Data Management and Sharing: https://grants.nih.gov/grants/guide/notice-files/NOT-OD-21-013.html). While several data resources listed in Table 2 provide data already harmonized and ready to use in analysis, many of the data resources listed are opportunities to discover and request original data only. Investigators requesting data will very often need to harmonize the data themselves, yet without access to ready guidance as to how to carry out and report the retrospective harmonization according to best practices in the field, especially across the multiple types of data ADRD population neuroscientists work with. This is a recipe for an “anything goes” approach, and it has been shown that harmonization and reporting practices vary widely across studies (Fortier et al., 2017). We hope this Tutorial will help begin to fill this gap. Finally, we recommend that new and existing investigators help develop further best practices and training materials for our field to standardize and enhance rigor across approaches.

## Figures and Tables

**TABLE 1:** Core competencies/gaps in knowledge and specific training resources in harmonization of population neuroscience studies of ADRD.

Core competency/gap in knowledge	Training resource(s)
**Overall harmonization skills**	
Multidisciplinary collaboration skills	• Scientific leadership and development courses; refer to researcher’s own institution offerings on these topics ◦ Example at the University of Pittsburgh: https://www.oacd.health.pitt.edu/micro-credential-postdocs; Note: Although it is aimed at postdocs, faculty may also enroll
	• Mentor(s) with multidisciplinary project leadership experience ◦ Discuss during mentorship meetings
	• Mentors or consultants with specific area expertise ◦ One-on-one and/or lab meetings ◦ Directed readings
Pre-statistical harmonization and detailed documentation of study design, variables, and variable transformation	• Maelstrom retrospective harmonization guidelines (Fortier et al., 2017)
	• Common data element resources on how variables can be mapped to a harmonized variable ◦ NIH’s common data element (CDE) repository (https://cde.nlm.nih.gov/) ◦ Gateway to Global Aging Data site’s (https://g2aging.org/documentation) data documentation
	• Considerations for harmonization, pooled study design, and analyses (Lesko et al., 2018)
Reproducible statistical coding, analysis, and power calculations	• Statistical coding and analysis coursework at researcher’s own institution
	• GitHub for promoting code reproducibility (https://github.com/)
	• Learn about incorporating code review (Vable et al., 2021)
	• Mentor(s) with biostatistics expertise
	• Online coursework
	• LinkedIn Learning (see if your institution has an institutional subscription)
	• Statistical Horizons (https://statisticalhorizons.com/) and Code Horizons (https://codehorizons.com/)
	• Neuroimaging analysis in R through Neuroconductor (https://neuroconductor.org/courses)
**Demographic and clinical variable harmonization skills**	
Domain expertise	• Mentors or consultants with domain expertise ◦ One-on-one and/or lab meetings ◦ Directed readings
**Neurocognitive assessment harmonization skills**	
Domain expertise	• A mentor or consultant who is a neuropsychologist ◦ One-on-one and/or lab meetings ◦ Directed readings
	• Observation of neuropsychological test administration and scoring
	• Review of test protocols, materials, and stimuli
	• Suggested readings ◦ Common tests selected for data sharing in AD research, characteristics, considerations (Bellio et al., 2020)
Methods expertise such as standardization, equipercentile equating, multiple imputation, factor analysis, and item response theory-based approaches	• A mentor or consultant who has psychometric methods expertise ◦ One-on-one and/or lab meetings ◦ Directed readings
	• Advanced Psychometric Methods in Cognitive Aging Research (*Ψ*MCA: https://psymca.org/) Annual conference and workgroups. Workgroups are application-based admission.
	• Suggested readings ◦ Methods to harmonize and combine neuropsychological assessment data for meta-analysis (Griffith et al., 2015) ◦ Cautions about sum and mean score approaches (standardization) (McNeish and Wolf, 2020) ◦ Multiple imputation approach to harmonization in AIBL and ADNI (Shishegar et al., 2021) ◦ Equipercentile equating based approach in the NACC Uniform Data Set neuropsychological test battery (Monsell et al., 2016) ◦ Detailed paper with workflow on IRT-based neuropsychological data harmonization and co-calibration in studies of cognitive aging and ADRD (Mukherjee et al., 2022) ◦ IRT-based harmonization of neuropsychological data for an analysis of genetics in late-onset AD subgroups across five studies (Mukherjee et al., 2020) ◦ Example cross-national harmonization (US and India) (Vonk et al., 2022) ◦ Cross-national harmonization with brief cognitive assessments, with good discussion of assumptions, alternatives (Kobayashi et al., 2021)
**Neuroimaging harmonization skills**	
Image processing skills	• A mentor or consultant who has neuroimaging harmonization expertise ◦ One-on-one and/or lab meetings ◦ Directed readings
	• FreeSurfer neuroimaging processing course: https://surfer.nmr.mgh.harvard.edu/fswiki/CourseDescription
	• Oxford Centre for Functional Magnetic Resonance Imaging of the Brain (FMRIB) Software Library (FSL) course and online resources: https://open.win.ox.ac.uk/pages/fslcourse/website/
	• NIPY: Neuroimaging analysis using Python https://nipy.org/#
	• Neurohackademy Lectures: https://neurohackademy.org/course_type/lectures/
	• PET Pharmocokinetics Course: A 3-day course which provides an overview of principles involved in PET kinetic modeling and analysis. The course includes lectures, interactive discussions, and hands-on computer exercises. It runs every other year before NeuroReceptor Mapping (NRM) and before Brain on the intervening years
	• Rotations in your local MRI and/or PET imaging center
Pre- and post-statistical harmonization	• See *Statistical coding and analysis* under *Overall harmonization skills* above
	• Suggested readings ◦ Original ComBat paper for gene expression microarray data (Johnson et al., 2007) ◦ MRI, cross-sectional regression-based harmonization ◦ White Stripe image-intensity normalization (Shinohara et al., 2014) ◦ RAVEL (Removal of Artificial Voxel Effect by Linear regression) (Fortin et al., 2018) ◦ ComBat for cortical thickness (Fortin et al., 2016) ◦ ComBat for diffusion tensor imaging (Fortin et al., 2017) ◦ ComBat for fMRI (Yu et al., 2018) ◦ ComBat combined with generalized additive models (ComBat-GAM) to address harmonization across a wide age range (Pomponio et al., 2020) ◦ CovBat to address site effects in covariance (in addition to the more typical site effects in mean and variance) (Chen et al. 2022) ◦ MRI, longitudinal regression-based harmonization ◦ ComBat for longitudinal cortical thickness (Beer et al., 2020) ◦ Machine learning-based harmonization ◦ MRI harmonization via MISPEL (Multi-scanner Image harmonization via Structure Preserving Embedding Learning) when more than two scanners are used (Torbati et al., 2021b) ◦ DeepHarmony addresses MRI contrast differences across two scanners (Dewey et al., 2019) ◦ mica addresses MRI contrast differences across more than two scanners (Wrobel et al., 2020) ◦ PET harmonization ◦ Standardization with Centiloids for PET amyloid imaging (Klunk et al., 2015; Royse et al., 2021) ◦ Non-linear distributional mapping (NoDiM) to address potential non-linearities in amyloid PET tracer measurement scales (Properzi et al., 2019) ◦ Impact of RAVEL on MRI and PET outcomes (Minhas et al., 2020) ◦ New and combined pipelines ◦ Combining RAVEL and ComBat to harmonize across different scanner strengths and remove both variation due to varying imaging intensity and other scanner effects (Torbati et al., 2021a)

This table should be used by the researcher seeking training to identify the core competencies which are knowledge gaps for them in the left column. The researcher should then select one or more relevant training resources in the right column to address the training needs.

**TABLE 2:** Data resources for harmonization of population neuroscience studies of ADRD.

Study or consortium and description	Location
**Sources of multiple studies that could be harmonized together**	
**ADDI: Alzheimer’s Disease Data Initiative.** A source for data sharing and funding with data analysis tools.	• https://www.alzheimersdata.org/
**AD Knowledge Portal.** An open access data repository established as part of the AMP-AD program (Accelerating Medicines Partnership in Alzheimer’s Disease). Shares National Institute on Aging (NIA)-funded translational data in the cognitive aging-to-ADRD spectrum.	• https://adknowledgeportal.synapse.org/
**DPUK: Dementias Platform UK.** A partnership of public and private organizations based at Oxford University. A source of shared data from 42 cohorts and more than 3 million study participants through their data portal.	• https://www.dementiasplatform.uk/
	• Data portal: https://www.dementiasplatform.uk/research-hub/data-portal
• DPUK Paper: (Bauermeister et al., 2020)
**GAINN: Global Alzheimer’s Association Interactive Network.** A source of information on shared datasets with tools for cohort discovery (which cohorts could be used to answer your scientific question of interest).	• https://gaain.org/
	• GAINN Paper: (Ashish et al., 2016)
**Human Connectome Project (HCP).** A source of data across 20 studies of brain connectomics.	•https://www.humanconnectome.org/
**Rush Alzheimer’s Disease Center.** A source of data across multiple Rush cohort studies. Includes: clinical evaluations, cognitive testing, laboratory tests, neuroimaging, etc.	• https://www.radc.rush.edu/
	• Religious Orders Study (ROS) Paper: (Bennett et al., 2012a)
	• Rush Memory and Aging Project (MAP) Papers: (Bennett et al., 2005, 2012b)
	• Rush Minority Aging Research Study (MARS) Paper: (Barnes et al., 2012)
**Sources of single studies that could be used in a new retrospective data harmonization across studies**	
**ADNI: Alzheimer’s Disease Neuroimaging Initiative.** Multicenter study with data from participants across the AD spectrum. Clinical, neuropsychological, and neuroimaging data.	• https://adni.loni.usc.edu/
	• ADNI Papers: (Weiner et al., 2010, 2015, 2017)
**Cambridge Centre for Ageing Neuroscience (Cam-CAN).** A study of successful cognitive aging across a wide age range (18–87) with demographic, physiological, neuropsychological, and multimodal neuroimaging data. The neuroimaging study is a sub-study of a larger, population-based study.	• https://camcan-archive.mrc-cbu.cam.ac.uk//dataaccess/
	• Cam-CAN data paper: (Taylor et al., 2017)
**Harvard Aging Brain Study (HABS).** A longitudinal study of healthy cognitive aging vs. preclinical AD with clinical, neuropsychological, and multimodal neuroimaging data.	• https://habs.mgh.harvard.edu/researchers/
	• HABS data paper: (Dagley et al., 2017)
**Health and Retirement Study (U.S.).** A population representative survey on aging. May be useful for external validity assessment. Includes data on demographics, health (including cognitive data), healthcare services, work and employment, economic status, family structure, and social network retirement.	• https://hrs.isr.umich.edu/about
**NACC: National Alzheimer’s Coordinating Center.** Multicenter study with data from more than 45,000 participants from the United States National Institute on Aging funded Alzheimer’s Disease Research Centers. Includes resources for trainees in their Research Education Component information (2^nd^ link).	• https://naccdata.org/
	• Research Education Component (REC) training resources: https://naccdata.org/adrc-resources/rec-home
	• NACC Papers: (Beekly et al., 2004, 2007)
**OASIS-3.** A longitudinal study of cognitive aging to dementia spectrum from the Washington University Knight Alzheimer Disease Research Center. As such, some of this data is likely part of the NACC data set (see above). Includes clinical, neuropsychological, and multimodal neuroimaging data.	• https://www.oasis-brains.org/
	• OASIS-3 data paper: (LaMontagne et al., 2019)
**PResymptomatic Evaluation of Experimental or Novel Treatments for AD (Prevent AD).** A longitudinal study of cognitively unimpaired people with a family history of AD. Includes clinical, neuropsychological, biofluid, and neuroimaging data.	• Neuroimaging data: https://openpreventad.loris.ca/
	• Other data: https://registeredpreventad.loris.ca/
	• Prevent AD data paper: (Tremblay-Mercier et al., 2021)
**Projects with retrospective data harmonization actively in progress or data that has already been harmonized; may also have additional data for new harmonization across studies**	
**CCC: Cross-Cohort Collaboration Consortium.** A collaborative across multiple observational cohort studies.	• https://chs-nhlbi.org/node/6539
**CHARGE: Cohorts for Heart and Aging Research in Genomic Epidemiology.** A source of genomic, risk factor, subclinical disease, and cardiovascular events data across multiple cohorts and more than 50,000 study participants. The NeuroCHARGE work group heads up collaborations with other consortia using cognitive, neuroimaging, and clinical neurological data.	• https://web.chargeconsortium.com/
	• CHARGE Paper: (Psaty et al., 2009)
**COSMIC: Cohort Studies of Memory in an International Consortium.** A source of data from 47 population-based cohort studies of cognitive aging from 35 countries (~150,000 study participants).	• https://cheba.unsw.edu.au/consortia/cosmic
	• COSMIC Paper: (Sachdev et al., 2013)
**ENIGMA: Enhancing Neuroimaging Genetics Through Meta Analysis.** An international collaboration of studies evaluating genetics in multiple brain-related conditions. May be a source of data, training materials.	• https://enigma.ini.usc.edu/
	• Videos including training presentations: https://bit.ly/3lHzDiw
	• ENIGMA Paper: (Stein et al., 2012)
**Gateway to Global Aging Data.** A source of cohort and data documentation, questionnaires, and harmonized longitudinal data from the Health and Retirement Studies from around the world (more than 40 countries). Includes data on demographics, health (including cognitive data), healthcare services, work and employment, economic status, family structure, and social network retirement.	• https://g2aging.org/
	• The U.S. Health and Retirement Study is one contributing study included on this site.
**National Institute on Aging Genetics of Alzheimer’s Disease Data Storage Site (NIAGADS).** Harmonized genomic and clinical data from 30^+^ cohort studies of Alzheimer’s disease. Endophenotype harmonization (e.g., cognition, brain MRI, amyloid PET imaging, autopsy measures of neuropathology, vascular risk factors, and fluid biomarkers) was recently funded and will be released in phases over the next 5 years. Through U24 AG074855, *Alzheimer’s Disease Sequencing Project Phenotype Harmonization Consortium*	• https://dss.niagads.org/
	• Studies included: https://dss.niagads.org/studies/
	• Future summaries of endophenotype harmonization: https://www.vmacdata.org/
	• Future home of harmonized images: https://loni.usc.edu/

## Data Availability

The original contributions presented in the study are included in the article/supplementary material, further inquiries can be directed to the corresponding author/s.
